# Virtual Reality-Based Rehabilitation in Children and Adolescents with Muscular Dystrophy: A Systematic Review of Feasibility, Engagement, and Clinical Outcomes

**DOI:** 10.3390/children13070895

**Published:** 2026-07-03

**Authors:** Insu Choi, Hwa Jin Cho, Song-Ai Kang, Won-Jae Kim, Min-Keun Song

**Affiliations:** 1 Department of Pediatrics, Chonnam National University Children’s Hospital, Chonnam National University Hospital, Chonnam National University Medical School, Gwangju 61469, Republic of Korea; insuchoi@jnu.ac.kr (I.C.); mdchohj@jnu.ac.kr (H.J.C.); 2 Department of Physical & Rehabilitation Medicine, Chonnam National University Hospital, Chonnam National University Medical School, Gwangju 61469, Republic of Korea; medlar01@jnu.ac.kr (S.-A.K.); dnsfla1206@naver.com (W.-J.K.)

**Keywords:** virtual reality, muscular dystrophy, pediatric rehabilitation, systematic review, stage-based framework

## Abstract

Background/Objectives: Muscular dystrophies (MDs) are progressive neuromuscular disorders in which rehabilitation is central to management, yet conventional physical therapy in children is constrained by motivation, accessibility, and the need to adapt across disease stages. Virtual reality (VR) offers an interactive, adaptable, and home-deliverable alternative, but prior reviews focused narrowly on upper-limb outcomes in Duchenne muscular dystrophy or on motor-learning paradigms. We aimed to evaluate VR-based rehabilitation in children and adolescents with MD across feasibility/adherence, engagement and psychological outcomes, and clinical motor outcomes, and to propose a stage-based conceptual framework. Methods: PubMed, Embase, and Cochrane CENTRAL were searched on 21 April 2026, following PRISMA 2020 (PROSPERO CRD420261380539). Eligible studies enrolled children or adolescents (mean age ≤ 18 years or separable pediatric data) with any MD who received VR/AR/MR/exergame/serious-game rehabilitation. Risk of bias was assessed with RoB 2.0 and ROBINS-I, and certainty with GRADE. Given substantial heterogeneity, findings were synthesized narratively by disease stage. Results: Eight studies (2017–2024; 221 participants) met the inclusion criteria. No serious VR-related adverse events occurred, and feasibility and tolerability were consistently favorable. Engagement and psychological outcomes showed favorable trends, including sustained motivation and reduced perceived fatigue. Clinical motor outcomes were heterogeneous and stage-dependent. Conclusions: The evidence base is limited and clinically heterogeneous, precluding meta-analysis, with Low GRADE certainty for feasibility, safety, and adherence and Very low for the remaining four domains. Key limitations include small sample sizes, substantial clinical and methodological heterogeneity, and only a single advanced-stage study. The findings provisionally support a stage-dependent role for VR-based rehabilitation in pediatric MD: motor training in the ambulatory stage, upper-limb maintenance and interface-adapted training in the transitional stage, and feasibility-, engagement-, and psychological-support applications in advanced disease. Stage-stratified trials with standardized, domain-specific outcomes and explicit virtual-to-real transfer assessment are warranted.

## 1. Introduction

### 1.1. Disease Background and Clinical Burden

Muscular dystrophies (MDs) comprise a heterogeneous group of inherited neuromuscular disorders characterized by progressive skeletal muscle degeneration and weakness [1]. More than 30 distinct subtypes have been identified, each associated with specific patterns of muscle involvement and underlying genetic abnormalities [1]. Among these disorders, Duchenne muscular dystrophy (DMD) represents the most prevalent and severe phenotype, affecting approximately 1 in 3500 to 6000 live male births worldwide [2]. DMD is caused by mutations in the dystrophin gene located on the X chromosome (Xp21), resulting in absent or markedly reduced dystrophin expression. Dystrophin is a critical structural protein required for the preservation of muscle fiber membrane integrity [1]. Deficiency of dystrophin leads to progressive muscle fiber degeneration and subsequent replacement of muscle tissue with fibrofatty tissue [2], ultimately resulting in irreversible functional deterioration [3].

Clinically, DMD typically presents in early childhood, most commonly between 2 and 5 years of age, with early involvement of the pelvic and scapular girdle musculature manifesting as gait difficulty, frequent falls, and delayed motor milestone acquisition [4]. In the corticosteroid-treatment era, contemporary natural history studies indicate that loss of ambulation occurs on average in the early teens, with up to 30% of patients becoming non-ambulant by 10 years and up to 90% by 15 years of age [4]. Loss of ambulation is subsequently followed by progressive upper-extremity dysfunction, scoliosis, respiratory insufficiency, and cardiomyopathy during advanced disease stages [3]. Although respiratory failure was historically the principal cause of mortality, advances in corticosteroid therapy, spinal stabilization, and assisted ventilation have prolonged survival into the third and fourth decades of life, such that cardiomyopathy has now emerged as a leading cause of death [5]. Becker muscular dystrophy (BMD), a milder allelic variant of DMD, follows a comparable but more slowly progressive disease course, with onset typically occurring during adolescence and substantial variability in clinical severity [5]. Collectively, MDs impose a considerable burden on affected individuals, caregivers, and healthcare systems through both direct medical expenditures and profound reductions in quality of life [3].

At present, no definitive curative therapy exists for MDs [6]. Clinical management therefore remains primarily supportive and multidisciplinary, encompassing corticosteroid therapy, cardiopulmonary surveillance, nutritional support, and orthopedic management [6]. Rehabilitation is widely recognized as a fundamental component of comprehensive care [6], with the principal objectives of preserving functional capacity, preventing contractures, and maximizing quality of life for as long as possible [3]. Given the progressive and stage-dependent nature of MDs, rehabilitation strategies must be continually individualized according to the patient’s evolving functional status, ranging from motor preservation during ambulatory stages to supportive and palliative interventions in advanced non-ambulatory disease [7].

### 1.2. Limitations of Conventional Rehabilitation

Conventional rehabilitation for pediatric MD commonly includes stretching exercises, aerobic conditioning, balance training, respiratory physiotherapy, and postural management [6]. Although these interventions may contribute to the preservation of motor function, several limitations constrain their long-term effectiveness and accessibility. First, because MDs are characterized by progressive muscle fragility, exercise regimens must be carefully calibrated to avoid overexertion, which may accelerate muscle injury [3], while simultaneously providing sufficient therapeutic stimulus to maintain functional capacity [6]. Second, motivational barriers are particularly significant in pediatric populations, in whom sustained participation in repetitive therapeutic exercises is often difficult to maintain without engaging in interactive intervention formats [8].

Access to specialized rehabilitation services also remains limited by geographical distance, transportation challenges, and the physical demands associated with travel, particularly among children with advanced motor impairment [7]. These barriers became substantially more pronounced during the COVID-19 pandemic, when many children with MD were unable to attend rehabilitation centers [9]. In a survey of 272 families conducted by Tunç et al., the majority of children reportedly received no physiotherapy during the pandemic, and only 6% had access to a home-based telerehabilitation program [9]. These findings underscore the urgent need for rehabilitation strategies that are accessible, home-based, and capable of sustaining patient engagement.

### 1.3. Virtual Reality as a Rehabilitation Tool

Virtual reality (VR) is an interactive technology that generates immersive three-dimensional environments through integrated visual, auditory, and, in certain systems, tactile feedback mechanisms [10]. Within rehabilitation settings, VR systems are generally classified as immersive, semi-immersive, or non-immersive, with commercial motion-capture platforms such as the Xbox Kinect and PlayStation EyeToy representing commonly used semi-immersive systems. These commercially available, non-head-mounted systems have predominated in rehabilitation practice and research because of their low cost, wide availability, and ease of integration into clinical and home settings, whereas fully immersive head-mounted displays have historically been less common in this population; accordingly, the studies included in the present review relied predominantly on these widely used platforms [11]. VR-based rehabilitation interventions have been implemented across diverse clinical populations, including individuals recovering from stroke [12], children with cerebral palsy, and patients with multiple sclerosis [11], with reported benefits involving motor performance, motivation, and patient engagement [11].

In pediatric MD, VR offers several potentially important therapeutic advantages. Its capacity to deliver personalized, adaptive, and task-specific training within an engaging virtual environment directly addresses the dual challenges of maintaining therapeutic effectiveness [10] and sustaining long-term participation in rehabilitation [8]. Importantly, VR systems can be modified to accommodate progressive functional decline by adjusting interface and interaction demands. For example, rehabilitation tasks may transition from whole-body movement paradigms in ambulatory patients to fine distal-movement interfaces, such as the Leap Motion controller, in individuals with advanced upper-extremity weakness [13]. De Freitas et al. demonstrated that individuals with DMD achieved significantly superior performance using the Leap Motion interface compared with touch-screen or Kinect-based systems, likely reflecting relative preservation of distal finger function in this population and underscoring the importance of interface selection in VR-based rehabilitation [13].

VR rehabilitation is also highly compatible with telerehabilitation delivery models, permitting home-based intervention through video-call platforms and commercially available devices [9]. Telerehabilitation has been proposed as a feasible and effective strategy for children with neuromuscular disorders because it may reduce travel-related burden, improve access to specialized care, and support continuity of rehabilitation services [14]. In pediatric MD, where clinic attendance is frequently inconsistent because of physical limitations and geographic barriers, integration of VR into telerehabilitation frameworks may provide an important mechanism for reducing disparities in access to care. Beyond physical rehabilitation, VR-based interventions may also address psychological domains, including anxiety, diminished motivation, and fatigue perception, all of which are highly prevalent in children with MD and remain comparatively underrepresented in conventional rehabilitation research.

### 1.4. Evidence Gap and Rationale for This Review

Despite increasing interest in VR-based rehabilitation for pediatric MD, the available evidence remains limited and has not been comprehensively synthesized within a stage-based clinical framework. A systematic review by Baeza-Barragán [14] evaluated the effectiveness of VR in upper-extremity rehabilitation for DMD, identifying seven studies published between 2009 and 2019 and reporting improvements in functionality, quality of life, and motivation. However, that review was restricted primarily to upper-extremity outcomes and did not incorporate telerehabilitation interventions or broader MD phenotypes]. More recently, Kiper et al. [15] reviewed VR interventions in MD rehabilitation from a motor-learning perspective, including seven studies involving 440 individuals with DMD, and concluded that VR-based interventions may exert favorable effects on motor learning and performance. Although these reviews substantially advanced the field, neither conceptualized VR as a rehabilitation modality whose primary clinical role may evolve across the disease trajectory.

Since 2019, additional studies have expanded the application of VR to telerehabilitation settings, incorporated engagement and psychological outcome domains, and explored advanced disease stages in which restoration-oriented motor goals may no longer be realistic. Accordingly, the present review extends the existing literature in three principal ways: (i) inclusion of recent intervention studies published between 2017 and 2024 spanning ambulatory, transitional, and advanced disease stages; (ii) explicit differentiation between clinical rehabilitation evidence and motor-learning paradigm evidence, recognizing the differing inferential implications of these study designs; and (iii) application of a stage-based conceptual framework as the primary organizational structure for evidence synthesis.

### 1.5. Objectives

The objectives of this systematic review were: (1) to identify and characterize published studies evaluating VR-based rehabilitation interventions in children and adolescents with muscular dystrophy; (2) to synthesize evidence regarding the feasibility, safety, and adherence associated with VR-based rehabilitation in this population; (3) to evaluate the effects of VR-based rehabilitation on engagement and psychological outcomes, including motivation, sustained participation, fatigue perception, and anxiety, as well as clinical motor outcomes; and (4) to propose a stage-based conceptual framework describing the evolving clinical role of VR rehabilitation across the disease trajectory in pediatric muscular dystrophy.

## 2. Methods

### 2.1. Protocol and Registration

This systematic review was conducted and reported in accordance with the Preferred Reporting Items for Systematic Reviews and Meta-Analyses (PRISMA) 2020 statement [16]. The review protocol was developed a priori and registered with the International Prospective Register of Systematic Reviews (PROSPERO; registration ID CRD420261380539).

### 2.2. Eligibility Criteria

Studies were considered eligible if they satisfied the following criteria.

Population: children or adolescents with any form of muscular dystrophy (search terminology encompassing DMD, BMD, facioscapulohumeral, limb-girdle, congenital, Emery–Dreifuss, and myotonic dystrophy is provided in Supplementary Material S1), with a mean age ≤ 18 years or separable pediatric subgroup data within mixed-age cohorts.

Intervention: rehabilitation interventions incorporating virtual reality, augmented reality, mixed reality, exergames, serious games, or commercial motion-capture gaming systems.

Comparison: any rehabilitation comparator, including standard physical therapy, usual care, active control interventions, or within-subject pre–post and paired-baseline comparisons.

Outcomes: at least one quantitative outcome related to motor function, upper-extremity function, activities of daily living, quality of life, adherence, motivation, or safety.

Studies were excluded if the intervention lacked a virtual-environment or interactive-task component; if VR was used exclusively for non-rehabilitation purposes, including assessment, education, or pain management; if pediatric MD subgroup data could not be separated from mixed-age cohorts; if the publication was not available in English; or if the full text could not be retrieved.

### 2.3. Information Sources and Search Strategy

A systematic literature search was conducted on 21 April 2026, across three electronic databases: PubMed, Embase, and the Cochrane Central Register of Controlled Trials (CENTRAL). The search strategy incorporated both controlled vocabulary and free-text terminology spanning four conceptual domains: (1) disease-related terms (e.g., “muscular dystrophy,” “Duchenne,” “Becker,” “myotonic dystrophy,” “facioscapulohumeral,” and “limb-girdle”); (2) pediatric population terms (e.g., “child,” “adolescent,” and “pediatric”); (3) rehabilitation-related terms (e.g., “rehabilitation,” “physiotherapy,” “physical therapy,” and “respiratory rehabilitation”); and (4) VR-related technology terms (e.g., “virtual reality,” “exergame,” “serious game,” “Kinect,” “Wii,” and “gamification”). Complete search strategies for each database are provided in Supplementary Material S1. No language restrictions were applied during the initial search process; restriction to English-language publications was implemented during eligibility assessment.

All retrieved records were imported into EndNote (Clarivate Analytics) for reference management. A total of 67 records were identified across the three databases (PubMed n = 11, Embase n = 39, Cochrane CENTRAL n = 17). Automated duplicate detection identified and removed 5 duplicate records, yielding 62 unique citations for title and abstract screening. To enhance the comprehensiveness of the search, the reference lists of all included studies and of relevant prior systematic reviews [14,15] were manually screened (backward citation searching). This process identified two additional candidate records; one met all eligibility criteria and was included [17], whereas the other was excluded at full-text assessment because its intervention lacked a virtual-environment or interactive-task component.

### 2.4. Study Selection and Data Extraction

**Figure 1:**
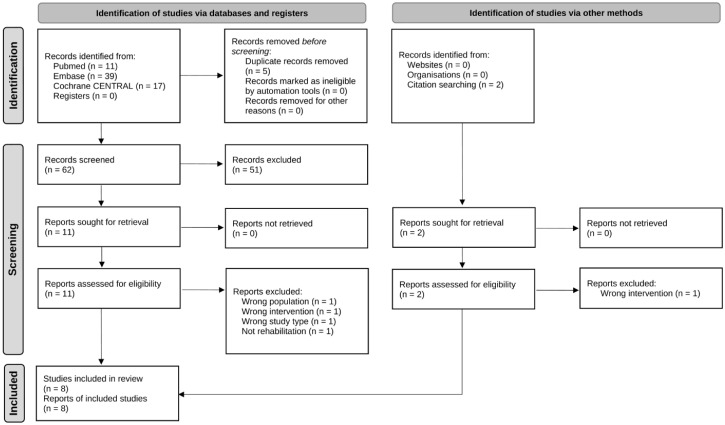
PRISMA 2020 flow diagram of study selection. The diagram illustrates the flow of records from database identification (n = 67; 5 duplicates removed in EndNote, yielding n = 62 unique records) through title and abstract screening (51 excluded), full-text retrieval (n = 11), and full-text exclusion with reasons (n = 4: 1 wrong population, 1 wrong intervention, 1 wrong study type, and 1 not rehabilitation). An additional study (Quadrado 2019 [17]) was identified through backward citation searching of the reference lists of included studies and relevant prior reviews, yielding 8 studies in the final synthesis. Included studies were categorized as multi-session clinical rehabilitation studies (n = 3: Heutinck 2018 [18], Baeza-Barragán 2023 [19], and Kurt-Aydin 2024 [20]) or narrative mechanistic/feasibility studies (n = 5: Al-Mfarej 2023 [21], de Freitas 2019 [13], Capelini 2017 [22], Massetti 2018 [23], and Quadrado 2019 [17]). Title and abstract screening was independently performed by 2 reviewers (Cohen’s κ = 0.897, indicating almost perfect agreement).

Title and abstract screening was independently conducted by two reviewers according to the predefined eligibility criteria. Inter-rater agreement was 96.8% (Cohen’s κ = 0.897, indicating almost perfect agreement), and the two disagreements were resolved through consensus discussion. Full-text articles were retrieved for all studies meeting initial screening criteria and independently evaluated by both reviewers, with reasons for full-text exclusion systematically documented. The study-selection process is summarized in the PRISMA 2020 flow diagram (Figure 1).

Data extraction was performed by one reviewer using a piloted and standardized extraction form that captured study characteristics, participant characteristics, intervention details, comparator characteristics, outcome measures, study results, adverse events, adherence outcomes, and risk-of-bias information. All extracted data were independently verified against the original source articles by a second reviewer, and discrepancies were resolved through consensus. The characteristics of the included studies are summarized in Table 1.

### 2.5. Risk-of-Bias Assessment

Risk of bias was evaluated using design-specific assessment tools. Randomized studies were assessed using the Cochrane Risk of Bias 2.0 (RoB 2.0) tool [24], which evaluates five methodological domains, whereas non-randomized studies were assessed using the Risk of Bias In Non-randomized Studies of Interventions (ROBINS-I) tool [25], which evaluates seven domains. For Kurt-Aydin 2024 [20], the randomized VR-versus-Biofeedback comparison and the non-randomized VR-versus-Control comparison were evaluated separately because each required application of a different risk-of-bias instrument; both assessments are presented in Table 2. For the two crossover randomized studies (de Freitas 2019 [13] and Massetti 2018 [23]), the RoB 2 adaptation for crossover trials was applied. Quadrado 2019 [17], in which participants were randomly allocated to button-press or gesture (virtual) task sequences, was likewise assessed using the RoB 2 crossover adaptation.

**Figure 2:**
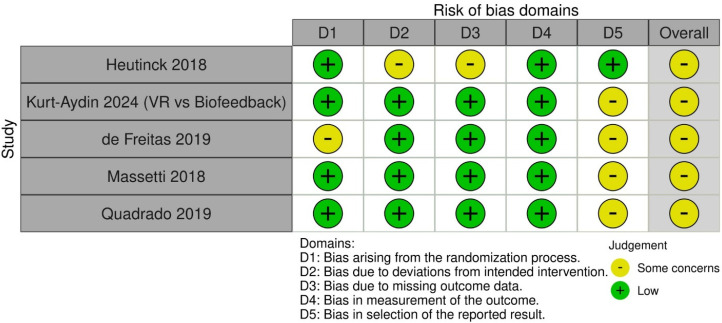
Risk-of-bias assessment for the 5 randomized study designs evaluated using RoB 2.0 [24] (Heutinck 2018 [18]; the randomized VR-versus-Biofeedback comparison in Kurt-Aydin 2024 [20]; de Freitas 2019 [13]; Massetti 2018 [23]; and Quadrado 2019 [17]). The traffic-light plot presents domain-level and overall risk-of-bias judgments for each randomized comparison. Symbols: green plus sign (+), Low risk; yellow minus sign (−), Some concerns; red multiplication sign (×), High risk. Visualization generated using the robvis tool [26].

**Figure 3:**
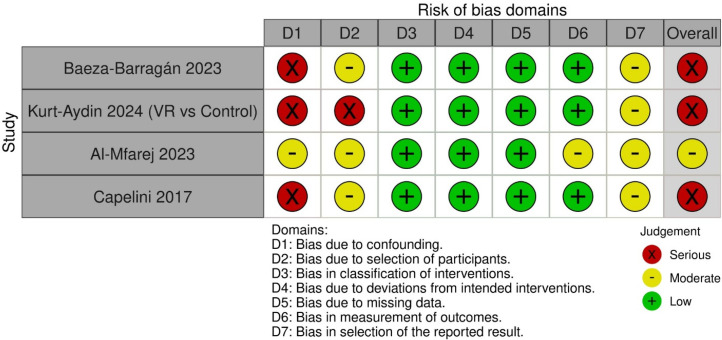
Risk-of-bias assessment for the 4 non-randomized study designs evaluated using ROBINS-I [25] (Baeza-Barragán 2023 [19]; the non-randomized VR-versus-Control comparison in Kurt-Aydin 2024 [20]; Al-Mfarej 2023 [21]; and Capelini 2017 [22]). The traffic-light plot presents domain-level and overall risk-of-bias judgments for each non-randomized comparison. Symbols: green plus sign (+), Low risk; yellow minus sign (−), Moderate risk; red multiplication sign (×), Serious risk. Critical risk and No information categories are included in the legend, although neither classification was assigned in the present review. Visualization generated using the robvis tool [26].

Risk-of-bias assessments were performed by one reviewer and independently verified by a second reviewer, with disagreements resolved through consensus discussion. Concordance for overall judgments was 100% (Cohen’s κ = 1.0). Results are summarized in Table 2 and presented graphically as ROBVIS traffic-light plots in Figure 2 (RoB 2.0) and Figure 3 (ROBINS-I).

### 2.6. Synthesis Methods

Quantitative pooling was prespecified as the preferred synthesis approach, but was ultimately not feasible because only one parallel-group trial provided fully extractable data for the calculation of between-group standardized effect estimates. Furthermore, the included studies demonstrated substantial clinical heterogeneity with respect to disease stage, intervention purpose, and comparator structure, as well as methodological heterogeneity in outcome time frames ranging from single-session assessments to 20-week interventions. Accordingly, the review was conducted as a systematic review with structured narrative synthesis.

**Figure 4:**
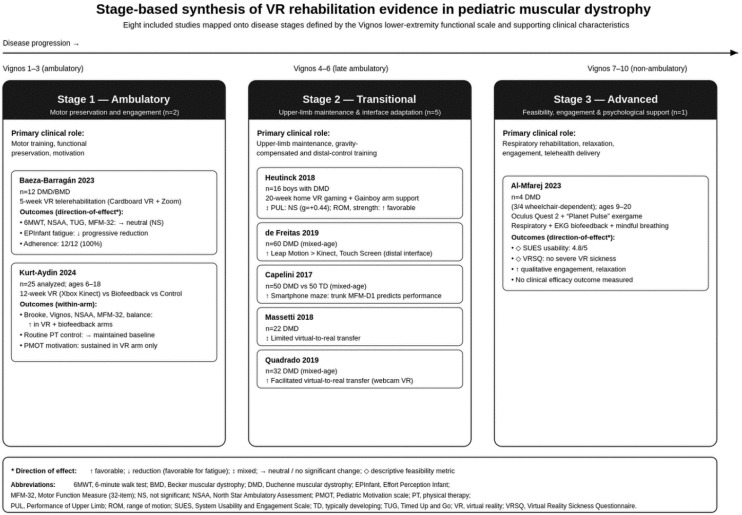
Stage-based synthesis framework for virtual reality rehabilitation in pediatric muscular dystrophy. The 8 included studies were mapped across 3 disease stages defined according to the Vignos lower-extremity functional scale and supporting clinical characteristics, including ambulatory status, age, and wheelchair dependency: Stage 1 (ambulatory; n = 2 studies; multi-session clinical rehabilitation studies with active comparators), Stage 2 (transitional/late-ambulatory to early non-ambulatory; n = 5 studies; the most data-rich stage, incorporating 1 parallel-group RCT and 4 motor-learning narrative studies), and Stage 3 (advanced; n = 1 feasibility study; representing the greatest evidence gap). The framework illustrates the clinical and methodological heterogeneity of the included studies and provides the rationale for the use of structured narrative synthesis rather than formal meta-analysis.

Direction of effect (favorable, neutral, mixed, unfavorable, or descriptive-only) was extracted for each study and outcome domain and summarized descriptively, with consideration of study size, design characteristics, and risk of bias. A three-stage disease framework (Stage 1: ambulatory/early; Stage 2: transitional/late-ambulatory to early non-ambulatory; Stage 3: advanced/multi-domain), based on the Vignos lower-extremity functional scale and supplemented by ambulatory status, age, and wheelchair dependency, served as the primary organizational framework for synthesis (Figure 4). To provide a descriptive statistical integration of effect magnitude without pooling, single-study standardized effect estimates (Hedges’ g with 95% confidence intervals) were calculated wherever the reported summary statistics permitted. In practice, sufficient data for between-group standardized effect estimates were available only for the parallel-group trial (Heutinck 2018 [18]), for which Hedges’ g values are reported in the synthesis and in Table 3; the remaining studies did not report the summary statistics (e.g., group means, standard deviations, and sample sizes for the relevant contrasts) required to compute comparable standardized effect sizes, and effect estimates were therefore neither calculated for those studies nor pooled across studies.

Certainty of evidence was evaluated at the outcome-domain level using the Grading of Recommendations Assessment, Development and Evaluation (GRADE) framework [27], with downgrading applied for risk of bias, inconsistency, indirectness, imprecision, and publication bias where appropriate. Final certainty assessments for each outcome domain are presented in Table 3.

## 3. Results

### 3.1. Study Selection and Characteristics

The systematic literature search identified 67 records (PubMed n = 11, Embase n = 39, Cochrane CENTRAL n = 17). Following duplicate removal (n = 5), title and abstract screening, and full-text eligibility assessment, 8 studies met the predefined inclusion criteria. The study-selection process, including reasons for full-text exclusion (n = 4), is summarized in the PRISMA 2020 flow diagram (Figure 1).

The 8 included studies were published between 2017 and 2024 and originated from 5 countries, collectively representing 221 participants with MD. Most participants had DMD, although BMD cohorts were also included in Baeza-Barragán 2023 [19] and Kurt-Aydin 2024 [20]. Participants were exclusively or predominantly male, consistent with the X-linked inheritance pattern of DMD and BMD. Four motor-learning studies (de Freitas 2019 [13]; Capelini 2017 [22]; Massetti 2018 [23]; Quadrado 2019 [17]) enrolled mixed-age DMD cohorts with a mean age ≤ 18 years but did not consistently report separable pediatric-specific outcomes.

Study designs included 1 parallel-group RCT (Heutinck 2018 [18]), 1 partially randomized three-arm trial (Kurt-Aydin 2024 [20]), 1 single-group A–B paired design study (Baeza-Barragán 2023 [19]), 4 single-session motor-learning paradigms, and 1 single-session feasibility study. Detailed characteristics of study populations, interventions, comparators, and VR platforms are summarized in Table 1.

### 3.2. Risk of Bias

Risk-of-bias assessments are summarized in Table 2 and presented graphically in Figure 2 and Figure 3. As detailed in Section 2.5, randomized designs were assessed with RoB 2.0 and non-randomized designs with ROBINS-I, with Kurt-Aydin 2024 contributing one assessment to each instrument [20].

All 5 RoB 2.0 assessments yielded an overall judgment of Some concerns, primarily reflecting limited reporting on deviations from intended interventions in open-label or home-based settings and multiple-comparison concerns in the single-session paradigms. Among the 4 ROBINS-I assessments, 3 were rated as Serious risk (Baeza-Barragán 2023 [19]; the VR-versus-Control comparison in Kurt-Aydin 2024 [20]; and Capelini 2017 [22]) and 1 as Moderate risk (Al-Mfarej 2023 [21]), with confounding (D1) representing the predominant source of bias across the non-randomized studies; in the VR-versus-Control comparison of Kurt-Aydin 2024 [20], non-randomized participant selection (D2) contributed an additional Serious-risk concern. Domain-level judgments for each study are presented graphically in Figure 2 (RoB 2.0) and Figure 3 (ROBINS-I).

Overall, no included study was judged to be at low risk of bias across all evaluated domains. The strongest comparative evidence was provided by Heutinck 2018 [18] and the randomized VR-versus-Biofeedback comparison in Kurt-Aydin 2024 [20], both of which received overall RoB 2.0 judgments of Some concerns. These methodological limitations supported the decision to use structured narrative synthesis rather than formal meta-analysis and directly informed the GRADE downgrading described in Section 3.4.

### 3.3. Stage-Based Synthesis of Outcomes

Consistent with the conceptual framework underpinning this review, findings are organized according to disease stage (ambulatory, transitional, and advanced), reflecting the evolving primary role of VR rehabilitation across the disease continuum. The complete stage-based synthesis framework is presented in Figure 4.

#### 3.3.1. Ambulatory Stage: Motor Preservation and Engagement

Two multi-session clinical studies contributed evidence relevant to the ambulatory stage: Baeza-Barragán 2023 [19] (single-group A–B paired design; n = 12; 5-week telerehabilitation intervention) and Kurt-Aydin 2024 [20] (3-arm partial RCT; n = 25 analyzed; 12-week clinic-supervised VR exercise program). In the active intervention arms of Kurt-Aydin 2024 [20], within-group functional improvements were observed across multiple motor domains, including the Brooke upper-extremity scale, Vignos lower-extremity scale, North Star Ambulatory Assessment (NSAA), Motor Function Measure (MFM-32), balance measures, and Timed Up and Go (TUG) performance. Comparable within-group improvements were also observed in the electromyography (EMG) biofeedback arm, whereas the routine physiotherapy control arm generally remained stable relative to baseline. Motivation assessed using the Pediatric Motivation Scale (PMOT) was sustained throughout the 12-week intervention exclusively within the VR group. In Baeza-Barragán 2023, no significant changes were observed in the primary outcome measure, the 6 min walk test (6MWT), or in secondary motor outcomes, including NSAA, TUG, and MFM-32, following 5 weeks of telerehabilitation. However, perceived fatigue, assessed using the Effort Perception Infant (EPInfant) scale, progressively decreased across intervention sessions despite gradual increases in exercise intensity.

#### 3.3.2. Transitional Stage: Upper-Limb Maintenance and Interface-Adapted Training

Heutinck 2018 [18] (parallel-group RCT with delayed-treatment crossover; n = 16) represented the only parallel-design study spanning the ambulatory-to-transitional disease stages. Following 20 weeks of home-based VR gaming combined with Gainboy^®^ dynamic arm support, the primary outcome, Performance of Upper Limb (PUL), demonstrated no significant between-group difference (g = +0.44, 95% CI [−0.56 to +1.44]). Nevertheless, significant favorable secondary effects were observed for elbow range of motion (g = +1.36, *p* = 0.018) and elbow extension strength (g = +0.94, *p* = 0.038). These findings suggest dissociation between component-level outcomes, including elbow range of motion and elbow extension strength, and the broader global PUL score. Specifically, VR-based gravity-compensated training appeared to improve targeted biomechanical domains without producing parallel improvements in overall function-level performance, potentially reflecting limited responsiveness of the global scale to the specific domain trained or incomplete translation of component-level gains into broader functional outcomes. Two single-session motor-learning studies (de Freitas 2019 [13]; Capelini 2017 [22]) provided indirect mechanistic evidence in mixed-age DMD cohorts spanning ambulatory and transitional disease stages. De Freitas 2019 [13] demonstrated that distal-control interfaces, particularly Leap Motion, outperformed full-body and touch-based interfaces in individuals with DMD, thereby supporting interface adaptation as an important transitional-stage rehabilitation principle. Capelini 2017 [22] further demonstrated that participants with DMD were able to acquire and retain performance on a smartphone-based marble-maze task, with trunk function (MFM-D1) emerging as a significant predictor of task performance. Quadrado 2019 [17] provided complementary transitional-stage evidence using a webcam-based virtual coincidence-timing task; individuals with DMD improved with practice and, notably, performance acquired in the virtual environment transferred to the real-environment task [17], in contrast to the limited virtual-to-real transfer subsequently reported by Massetti 2018 [23]. Taken together, these single-session studies indicate that virtual-to-real transfer in DMD is plausible but inconsistent, and appears sensitive to task design and difficulty.

#### 3.3.3. Advanced Stage: Feasibility, Engagement, and Psychological Support

Al-Mfarej 2023 [21] (single-session feasibility study; n = 4 participants with DMD, of 13 total participants [4] DMD and 9 neurotypical controls], including 3 of 4 who were wheelchair-dependent) was the only included study specifically targeting advanced-stage disease. The intervention consisted of a clinician-guided Oculus Quest 2 exergame (“Planet Pulse”) integrating respiratory and electrocardiographic biofeedback with deep-breathing and mindful-breathing exercises. The intervention demonstrated high usability (System Usability and Engagement Scale [SUES] score: 4.8/5), produced no severe VR-related sickness, and generated favorable qualitative feedback emphasizing engagement, relaxation, and breathing awareness. Although formal clinical efficacy outcomes were not assessed, these findings suggest that VR-based rehabilitation may remain feasible in advanced-stage MD as a platform for respiratory training, relaxation-oriented intervention, and psychological support in patients for whom restoration-oriented motor goals may no longer represent the primary therapeutic focus. Massetti 2018 [23] (single-session crossover RCT; n = 22) additionally provided evidence regarding cross-environment transfer. Although performance improved in both virtual and real conditions, transfer from the virtual environment to real-world performance was limited, highlighting the need for caution when interpreting the ecological validity and real-world generalizability of VR-trained motor skills.

#### 3.3.4. Outcomes Across Stages: Feasibility, Engagement, and Clinical Motor Patterns

Across all disease stages, no serious adverse events attributable to VR interventions were reported. Adherence was consistently high in the multi-session studies. Heutinck 2018 [18] reported a mean of 4.6 sessions per week relative to a prescribed frequency of 5 sessions weekly, corresponding to approximately 92% adherence across the 20-week intervention period. Kurt-Aydin 2024 [20] implemented a predefined adherence threshold of ≥80% and reported an attrition rate of 11% (3 of 28 participants), with withdrawals unrelated to the intervention itself. Baeza-Barragán 2023 [19] reported no attrition, with all 12 participants completing the 5-week telerehabilitation program. The home-based and telerehabilitation delivery models used in Heutinck 2018 [18], Baeza-Barragán 2023 [19], and Al-Mfarej 2023 [21] provide particularly relevant feasibility evidence for pediatric MD populations in whom access to clinic-based rehabilitation is frequently constrained by geographic and physical limitations. Engagement and psychological outcomes demonstrated a consistently favorable pattern across disease stages. In Kurt-Aydin 2024 [20], VR-based intervention uniquely sustained Pediatric Motivation Scale (PMOT) scores throughout the 12-week intervention period compared with electromyography (EMG) biofeedback. In Baeza-Barragán 2023 [19], perceived fatigue measured using the Effort Perception Infant (EPInfant) scale progressively declined across intervention sessions despite increasing exercise intensity. In advanced-stage participants from Al-Mfarej 2023 [21], high usability ratings and favorable qualitative themes related to relaxation, breathing awareness, and enjoyment were consistently reported. In contrast, clinical motor outcomes remained heterogeneous and appeared strongly influenced by disease stage and intervention target.

### 3.4. Summary by Outcome Domain (GRADE)

GRADE assessments across the 5 predefined outcome domains are summarized in Table 3. Certainty of evidence was rated as Very low for 4 domains, including motor function assessed using clinical scales, upper-extremity function, engagement and psychological outcomes, and motor learning. Certainty was rated as Low for feasibility, safety, and adherence. Across domains, the principal reasons for downgrading were imprecision and inconsistency, reflecting small sample sizes and substantial heterogeneity in outcome measures and intervention characteristics. Indirectness represented an additional concern within the motor-learning domain because performance during single-session experimental tasks may not directly correspond to clinically meaningful motor function. Certainty was rated as Low for feasibility, safety, and adherence, primarily reflecting the predominantly observational nature of the available evidence base. Publication bias was not formally evaluated because the limited number of included studies (k = 8) precluded meaningful funnel-plot analysis.

## 4. Discussion

### 4.1. Principal Findings

This review synthesized evidence from 8 studies evaluating VR-based rehabilitation in pediatric muscular dystrophy and identified a stage-dependent pattern of therapeutic roles and outcomes. Across the included studies, VR interventions demonstrated favorable feasibility and safety profiles, with high adherence rates and no reported serious adverse events. Engagement and psychological outcomes, when evaluated, consistently showed favorable trends. In contrast, clinical motor outcomes exhibited structured heterogeneity according to disease stage: the ambulatory stage was characterized primarily by signals of motor improvement within active intervention groups, the transitional stage by preservation of selected functional domains, and the advanced stage by emphasis on feasibility, engagement, relaxation, and psychological support. Collectively, these findings support a provisional conceptual reframing of VR in pediatric MD. Rather than functioning solely as a uniform motor-training modality, VR may fulfill distinct stage-dependent clinical roles in which the therapeutic emphasis progressively shifts from motor training toward engagement, supportive care, and psychological well-being as disease severity advances.

These findings extend prior systematic reviews in several important ways. Baeza-Barragán et al. [14] reported improvements in functionality, quality of life, and motivation associated with VR interventions in DMD; however, the evidence base available at that time was limited to studies published before 2019, and only 1 of the 7 included studies (Heutinck 2018 [18]) directly evaluated VR intervention efficacy in DMD, whereas the remaining studies primarily examined VR interfaces within motor-learning paradigms [14]. Kiper et al. [15] similarly focused on motor learning and concluded that VR-based interventions may exert favorable effects on motor learning and motor performance [15]. The present review expands upon this literature by incorporating studies published between 2017 and 2024, distinguishing clinical rehabilitation evidence from motor-learning evidence, extending the scope to include telerehabilitation and respiratory/psychological domains, and applying a stage-based framework as the primary organizational structure for synthesis.

### 4.2. Feasibility and Safety as the Strongest and Most Consistent Evidence

As detailed in Section 3.3, the most consistent finding across the included studies was the favorable feasibility and safety profile of VR-based rehabilitation in pediatric MD, with no serious intervention-related adverse events reported. This observation is particularly important given the physiological vulnerability of children with MD, including susceptibility to overexertion-related muscle injury, progressive weakness, and cardiopulmonary complications [6]. The overall safety profile observed across the included studies is also consistent with established exercise recommendations for DMD, which support moderate-intensity activity while discouraging excessive eccentric or high-intensity loading [6].

The telerehabilitation approaches implemented by Baeza-Barragán et al. [19], using Zoom and Cardboard VR glasses, and by Al-Mfarej et al. [21], using clinician-guided telehealth delivery, represent clinically meaningful developments within pediatric MD rehabilitation. Previous reviews have identified limited access to specialized rehabilitation services as a major barrier to continuity of care in pediatric MD [14], a problem that became particularly evident during the COVID-19 pandemic [9]. The demonstrated feasibility of home-based VR rehabilitation therefore addresses a clinically significant gap in care delivery and is consistent with the World Confederation for Physical Therapy’s endorsement of telerehabilitation as a strategy for improving accessibility and maintaining continuity of rehabilitation services [28].

### 4.3. Engagement and Psychological Outcomes: An Underexplored but Promising Domain

One of the most notable findings of this review was the consistently favorable direction of effect observed for engagement and psychological outcomes across studies evaluating these domains. Historically, rehabilitation research in MD has focused predominantly on upper-extremity function [14] and motor-learning paradigms [15] with comparatively limited attention devoted to motivation, fatigue perception, enjoyment, and psychological well-being. The motivational findings reported by Kurt-Aydin et al. [20] are particularly noteworthy because sustained participant motivation throughout the full 12-week intervention period was observed only in the VR group, despite comparable within-group motor improvements in both the VR and electromyography biofeedback groups. This pattern is consistent with self-determination theory models applied to pediatric rehabilitation, which emphasize autonomy, competence, and relatedness as key determinants of intrinsic motivation, all of which may be preferentially supported by interactive VR-based environments [8].

Baeza-Barragán et al. [19] additionally reported progressive reductions in perceived fatigue (EPInfant) despite gradual increases in exercise intensity across intervention sessions. Al-Mfarej et al. [21] further extended this psychological dimension into advanced-stage disease, demonstrating that a VR exergame integrating respiratory biofeedback and mindful-breathing exercises was perceived as highly relaxing and enjoyable even among severely impaired non-ambulatory participants. Collectively, these findings support the possibility that VR may serve an important supportive-care role in advanced-stage MD, particularly in clinical contexts where restoration-oriented motor rehabilitation is no longer the principal therapeutic objective.

### 4.4. Clinical Motor Outcomes: Heterogeneity Reflects Stage-Dependent Roles

Clinical motor outcomes demonstrated substantial heterogeneity; however, the observed heterogeneity followed a structured stage-dependent pattern. Signals of motor benefit were most apparent during the ambulatory stage, partial preservation of targeted functional domains characterized the transitional stage, and motor outcomes were no longer the primary therapeutic focus in advanced-stage disease.

Within the ambulatory stage, the active intervention groups in Kurt-Aydin et al. [20] demonstrated favorable within-group trends across multiple motor domains. These findings are broadly consistent with prior evidence regarding moderate-intensity exercise in early-stage DMD, which suggests that structured exercise interventions may improve strength and functional performance during the ambulatory period when residual regenerative capacity remains partially preserved [6]. The relative stability observed in the routine physiotherapy control group, contrasted with improvement signals in both the VR and biofeedback intervention groups, is also broadly consistent with the expected natural history of DMD and suggests that actively engaging rehabilitation paradigms, whether VR-based or biofeedback-based, may contribute to delaying functional decline during earlier disease stages.

Baeza-Barragán et al. [19] did not identify significant differences in motor outcomes between conventional physiotherapy and VR-based telerehabilitation. Nevertheless, this apparent null finding should be interpreted within the context of the expected natural history of ambulatory DMD, in which annual decline in 6MWT distance has been estimated at approximately 40–70 m in untreated ambulatory boys [29]. Maintenance of motor performance over a relatively short intervention interval may therefore still carry clinical relevance in progressive neuromuscular disease.

Within the transitional stage, Heutinck et al. [18] observed no significant improvement in the primary outcome measure (PUL), although favorable secondary effects were identified for elbow range of motion and elbow extension strength. The dissociation between component-level improvements and the broader global PUL score may indicate limited responsiveness of the global scale to the specific biomechanical domain targeted by the intervention, namely, proximal arm movement facilitated through dynamic arm support. The integration of Gainboy dynamic arm support with VR gaming also illustrates an important rehabilitation principle: optimization of VR interface design according to residual motor function. This interpretation is reinforced by de Freitas et al. [13], who demonstrated that interfaces leveraging preserved distal finger function, such as Leap Motion, outperformed full-body and touch-based interfaces in DMD.

Massetti et al. [23] reported limited transfer of motor gains from virtual environments to real-world task performance, indicating that improvements achieved during VR-based training may not uniformly translate into ecologically meaningful functional outcomes. In contrast, Quadrado et al. [17] found that a coincidence-timing task practiced in a virtual environment facilitated transfer to the real-world task when task difficulty was appropriately graded, suggesting that virtual-to-real transfer in DMD is achievable but task-dependent. Capelini et al. [22] further demonstrated that trunk function (MFM-D1) significantly predicted task performance, highlighting postural stability and seating support as clinically relevant determinants of VR engagement and task execution during transitional disease stages.

### 4.5. The Stage-Based Framework: A Conceptual Contribution

The principal conceptual contribution of this review is the proposal of a stage-based framework in which the primary clinical role of VR rehabilitation evolves across the pediatric MD disease continuum, shifting from motor-oriented training during the ambulatory stage to upper-extremity preservation and interface-adapted rehabilitation during the transitional stage, and ultimately toward engagement-focused and supportive-care applications in advanced-stage disease (Figure 4).

This framework aligns with the broader evolution of rehabilitation paradigms in progressive neuromuscular disorders, in which therapeutic priorities progressively transition from restoration and recovery toward preservation of function, adaptation, participation, and quality of life [6]. The framework also carries important implications for VR system design and clinical implementation. Ambulatory patients may derive the greatest benefit from full-body motion-capture systems, such as Xbox Kinect, that facilitate rhythmic lower- and upper-extremity movement training. In contrast, transitional-stage patients may require rehabilitation systems adapted to residual motor capacity, including gravity-compensating arm supports such as Gainboy or distal-function-oriented interfaces such as Leap Motion. Advanced-stage patients may benefit most from low-demand immersive VR environments emphasizing respiratory training, relaxation, and psychological support delivered through telehealth-based platforms. For direct clinical application, these observations can be distilled into a few practical recommendations, offered cautiously given the low certainty of the underlying evidence. First, in the ambulatory stage, clinicians may consider VR-based active gaming as an engaging adjunct to conventional therapy to support motivation and adherence while maintaining moderate exercise intensity. Second, in the transitional stage, the VR interface should be matched to residual motor function, favoring gravity-compensated proximal training or distal-control interfaces that exploit preserved hand function. Third, across all stages, but particularly in advanced disease, VR may be deployed through home-based telerehabilitation to improve access and to address engagement, fatigue, and psychological well-being when restoration-oriented motor goals are no longer the primary focus.

### 4.6. Strengths and Limitations

Strengths of this review include independent dual-reviewer screening with high inter-rater agreement (Cohen’s κ = 0.897), design-appropriate risk-of-bias assessment using RoB 2.0 and ROBINS-I with complete concordance on overall judgments, and consistent application of a pre-specified disease-stage framework across all included studies. The review additionally distinguishes clinical rehabilitation evidence from motor-learning evidence, an analytic separation that has not been explicitly incorporated in prior reviews addressing VR rehabilitation in MD.

Several limitations should also be acknowledged. The evidence base was limited by the small number of included studies (k = 8), small individual sample sizes, substantial heterogeneity across VR platforms, predominantly short intervention durations (including several single-session paradigms), and outcome measures, and the absence of long-term follow-up data, all of which precluded formal meta-analysis. Four motor-learning studies (de Freitas 2019 [13]; Capelini 2017 [22]; Massetti 2018 [23]; Quadrado 2019 [17]) included mixed-age cohorts (mean age ≤ 18 years) without separable pediatric-specific estimates and were therefore retained primarily as indirect mechanistic evidence. In addition, disease-stage classification necessarily remained approximate because some studies spanned stage boundaries, including Heutinck 2018, which incorporated both late-ambulatory and early non-ambulatory participants [18]. The included evidence was also dominated by DMD; only Baeza-Barragán 2023 [19] and Kurt-Aydin 2024 [20] included a minority of participants with BMD, and other muscular dystrophy subtypes were not represented, so the findings cannot be assumed to generalize across MD phenotypes, whose disease trajectories differ substantially. Furthermore, engagement, motivation, and fatigue outcomes were assessed almost exclusively through self-reported instruments, without corroborating objective physiological indicators (e.g., heart-rate variability, respiratory or electromyographic measures), which limit the objectivity of the psychological and fatigue findings. Finally, the advanced-stage domain was represented by only a single feasibility study, limiting the strength of conclusions that can currently be drawn for this stage of disease.

### 4.7. Implications for Future Research and Clinical Practice

The findings of this review have several implications for future research and clinical implementation. First, adequately powered randomized controlled trials incorporating stage-stratified participant selection are needed. Given the limited sample sizes of currently available studies, multicenter collaborative study designs will likely be necessary to achieve sufficient statistical power and improve generalizability. Second, future investigations should incorporate standardized and stage-appropriate outcome measures sensitive to the specific functional domains targeted by VR interventions. The discrepancy observed in several included studies between neutral primary outcomes (e.g., PUL and 6MWT) and favorable secondary outcomes (e.g., range of motion, strength, and fatigue perception) suggests that broad global functional scales may insufficiently capture targeted benefits achievable through domain-specific VR rehabilitation. Kiper et al. [15] similarly emphasized the need for outcome measures aligned with the specific training domains addressed by VR-based interventions.

Third, integration of physiological monitoring into VR rehabilitation platforms through wearable heart-rate sensors, respiratory monitoring systems, or electromyography biofeedback may facilitate real-time monitoring of exercise intensity and safety during VR intervention sessions, thereby addressing limitations identified across several included studies. Baeza-Barragán et al. [14] specifically recommended the incorporation of respiratory and cardiac variables capable of capturing metabolic effort during and following exercise, as well as a clearer definition of appropriate intervention intensity for individuals with severe fatigue or respiratory compromise. Immersive head-mounted display systems may additionally provide greater engagement and more naturalistic three-dimensional movement experiences than currently predominant non-immersive systems and therefore warrant further investigation in pediatric MD populations [15].

Fourth, psychological outcomes, including anxiety, fatigue, motivation, and quality of life, should be considered co-primary outcomes in future VR rehabilitation trials. This recommendation reflects the stage-dependent shift in the primary therapeutic value of VR identified in the present review. The high prevalence of anxiety and behavioral comorbidities in DMD [21], together with the favorable psychological responses observed in advanced-stage participants, further underscores the importance of systematically evaluating these domains. Fifth, future studies should explicitly pre-specify and evaluate virtual-to-real transfer effects, given the limited transfer observed by Massetti et al. [23]. Finally, group-based VR telerehabilitation models may offer additional advantages by enhancing social interaction, reducing isolation in advanced disease, and improving the cost-effectiveness and scalability of rehabilitation delivery [28].

## 5. Conclusions

Across 8 studies evaluating VR-based rehabilitation in pediatric muscular dystrophy, VR interventions consistently demonstrated favorable feasibility, safety, and engagement across the disease continuum. Clinical motor outcomes followed a stage-dependent pattern, with motor improvement signals predominating during the ambulatory stage, functional preservation characterizing the transitional stage, and feasibility and psychological benefit emerging as the principal themes in advanced-stage disease. Engagement and psychological outcomes were consistently favorable when assessed. Importantly, the certainty of evidence was low for feasibility, safety, and adherence and very low for all other outcome domains (GRADE); accordingly, these stage-dependent observations should be regarded as provisional and hypothesis-generating rather than as confirmation of clinical efficacy, and the apparent benefits should not be overinterpreted. Collectively, these findings support a stage-dependent role for VR rehabilitation in pediatric MD, in which the primary therapeutic emphasis progressively evolves from motor-oriented rehabilitation toward engagement, supportive care, and psychological well-being as disease severity advances.

## Figures and Tables

**Table 1:** Characteristics of included studies (n = 8). Summary of study characteristics, including author, year, country, study design, participant characteristics, VR platform, intervention duration and frequency, comparator, and primary outcome. Studies are ordered according to inferential weight, with clinical rehabilitation studies incorporating comparator groups presented first, followed by narrative mechanistic and feasibility studies.

Study	Country	Study Design	Participants	VR Platform	Intervention Details	Comparator	Primary Outcome
Heutinck 2018 [18]	Netherlands	Exploratory parallel-group RCT with delayed-treatment crossover	n = 16 boys with DMD (intervention n = 7, control n = 9); late-ambulatory to early non-ambulatory stage; ages 7–17 years	PlayStation II EyeToy with Gainboy^®^ dynamic arm support (100% horizontal-plane gravity compensation)	20-week home-based VR gaming intervention; mean 4.6 sessions/week; biweekly physiotherapist coaching visits	Usual care (delayed-treatment crossover after T2)	Change in Performance of Upper Limb (PUL) total score from T0 to T2 (week 22)
Baeza-Barragán 2023 [19]	Spain (multicenter)	Single-group A–B within-subject paired design (prospective quasi-experimental study)	n = 12 boys with DMD/BMD; Vignos score 2.08–2.25; NSAA > 20 (ambulatory)	Cardboard VR glasses delivered via Zoom (telerehabilitation)	5-week, 10-session multimodal intervention including aerobic exercise, stretching, breathing exercises, relaxation, mindfulness, and VR activities	Conventional physiotherapy phase (within-subject comparison preceding telerehabilitation phase)	Change in 6 min walk test (6MWT) distance following the 5-week telerehabilitation intervention
Kurt-Aydin 2024 [20]	Türkiye	Three-arm partial RCT (VR and biofeedback groups randomized; control group non-randomized)	Recruited n = 28; analyzed n = 25 (VR n = 9, biofeedback n = 8, control n = 8); ages 6–18 years; DMD/BMD	Xbox 360 Kinect with goal-oriented exercise games	12-week clinic-supervised VR exercise program; 30 min/session, 3 sessions/week	EMG biofeedback (RCT comparison); routine physiotherapy (non-randomized comparison)	No single predefined primary outcome; multiple co-primary functional and motivation outcomes assessed
Al-Mfarej 2023 [21]	United States	Single-session mixed-methods feasibility study (within-subject design)	n = 13 (DMD n = 4 [3 of 4 wheelchair-dependent]; neurotypical controls n = 9); ages 9–20 years	Oculus Quest 2 with custom “Planet Pulse” VR exergame; respiratory band and EKG biofeedback	One-hour clinician-guided VR session incorporating deep-breathing (respiratory training) and mindful-breathing (relaxation) exercises	None (within-subject pre/post survey design)	Feasibility and usability assessed using the VR Sickness Questionnaire (VRSQ) and System Usability and Engagement Scale (SUES)
de Freitas 2019 [13]	Brazil + UK	Single-session randomized crossover device-comparison RCT	n = 60 participants with DMD and n = 60 typically developing controls; mean age 16 years, range 9–34 years; Vignos 6–8 among 40/54 reported participants	Three VR interaction interfaces compared: Kinect, touch screen, and Leap Motion	10 s range-zone bubble-touching task performed across the three interfaces	Within-subject comparison across interfaces	Number of bubbles touched during acquisition, retention, and transfer blocks
Capelini 2017 [22]	Brazil + UK	Single-session case–control motor-learning study	n = 50 participants with DMD and n = 50 typically developing controls; mean age 17.1 years, range 10–34 years	Smartphone-based marble-maze tilt task	Acquisition, short-term retention, and transfer blocks were performed within-session	Typically developing matched controls	Movement time (seconds) during smartphone maze-task performance across experimental phases
Massetti 2018 [23]	Brazil	Single-session crossover RCT (within-subject virtual ↔ real comparison)	n = 22 participants with DMD; Group A mean age 14.8 years, Group B mean age 16.8 years	Kinect-based reach-to-target task	Acquisition, retention, and transfer blocks with paired virtual and real task conditions (within-subject design)	Real-environment task (within-subject crossover comparison)	Movement time (milliseconds) required to reach the target in virtual versus real environments
Quadrado 2019 [17]	Brazil	Single-session crossover RCT (button-press vs gesture/virtual sequences)	n = 64 (32 with DMD; 32 typically developing controls); DMD mean age 18 ± 5.1 years (range 12–32)	Webcam-based virtual environment (gesture interface) and keyboard button-press interface	Coincidence-timing task across acquisition, retention, and transfer blocks (speed-increase and environment-exchange transfers)	Within-subject comparison across interfaces (button-press vs gesture) and typically developing controls	Timing error (constant, absolute, and variable error) during the coincidence-timing task

**Table 2:** Risk-of-bias assessment for the included studies. Domain-level judgments and traffic-light visualizations were generated using RoB 2.0 [24] for randomized study designs (Heutinck 2018 [18]; the randomized VR-versus-Biofeedback comparison in Kurt-Aydin 2024 [20]; de Freitas 2019 [13]; Massetti 2018 [23]; and Quadrado 2019 [17]) and ROBINS-I [25] for non-randomized study designs (Baeza-Barragán 2023 [19]; the non-randomized VR-versus-Control comparison in Kurt-Aydin 2024 [20]; Al-Mfarej 2023 [21]; and Capelini 2017 [22]). For Kurt-Aydin 2024 [20], the randomized and non-randomized comparisons were evaluated separately because they required different risk-of-bias assessment tools. Domain-level judgments for each study are presented graphically in Figure 2 (RoB 2.0) and Figure 3 (ROBINS-I). NA, not applicable (single supervised session). For ROBINS-I, “Some concerns” corresponds to a Moderate rating.

Study	Tool	Selection/Random./Confounding	Deviations from Intervention	Missing Data	Outcome Measurement	Reported Result	Overall Judgment
Heutinck 2018 [18]	RoB 2	Low	Some concerns	Some concerns	Low	Low	Some concerns
Baeza-Barragán 2023 [19]	ROBINS-I	Serious	Moderate	Low	Low	Moderate	Serious
Kurt-Aydin 2024 [20]—VR vs Biofeedback	RoB 2	Low	Low	Low	Low	Some concerns	Some concerns
Kurt-Aydin 2024 [20]—VR vs Control	ROBINS-I	Serious	Low	Low	Low	Moderate	Serious
Al-Mfarej 2023 [21]	ROBINS-I	Moderate	NA	Low	Moderate	Moderate	Moderate
de Freitas 2019 [13]	RoB 2	Some concerns	Low	Low	Low	Some concerns	Some concerns
Capelini 2017 [22]	ROBINS-I	Serious	Low	Low	Low	Moderate	Serious
Massetti 2018 [23]	RoB 2	Low	Low	Low	Low	Some concerns	Some concerns
Quadrado 2019 [17]	RoB 2	Low	Low	Low	Low	Some concerns	Some concerns

**Table 3:** Certainty of evidence by outcome domain (GRADE). Five outcome domains were evaluated at the body-of-evidence level using the Grading of Recommendations Assessment, Development and Evaluation (GRADE) framework: motor function on clinical scales; upper-extremity function; engagement, motivation, and fatigue perception; feasibility, safety, and adherence; and motor learning/task-specific transfer. Initial certainty ratings were assigned according to study design and subsequently downgraded for risk of bias, inconsistency, indirectness, imprecision, and publication bias where appropriate. Final certainty was rated as Very low for 4 domains and Low for 1 domain (feasibility, safety, and adherence).

Outcome Domain	Contributing Studies (k)	Initial Certainty	Downgrade Factors	Final Certainty	Justification Summary
Motor function (clinical scales): 6MWT, NSAA, MFM-32, Brooke, Vignos, TUG, balance, strength	3 multi-session clinical rehabilitation studies (k = 3): Heutinck 2018 [18], Baeza-Barragán 2023 [19], Kurt-Aydin 2024 [20]	High (initially rated high; direct evidence derived from 1 RCT and non-randomized comparative studies)	−1 Risk of bias (Some concerns to Serious; Heutinck [18] limited by small sample size and incomplete blinding information; Baeza-Barragán [19] used a single-arm design without parallel control; Kurt-Aydin [20] included a non-randomized control group) −1 Inconsistency (heterogeneous intervention designs and outcome measures; no domain pooled across ≥ 2 directly comparable studies) −2 Imprecision (very small study populations [n = 12–25/study]; wide confidence intervals; most confidence intervals crossed zero, including primary outcomes) −1 Indirectness (mixed disease stages and heterogeneous outcome instruments)	Very low	Across multi-session rehabilitation studies, the direction of effect ranged from favorable to neutral, with findings reflecting either functional preservation or within-group improvement. However, no outcome domain was supported by ≥2 directly comparable studies, and confidence intervals remained wide. Effect estimates should therefore be interpreted descriptively rather than quantitatively.
Upper-limb function (PUL, ROM, HHD strength)	1 parallel-group RCT (k = 1): Heutinck 2018 [18]	High (single parallel-group RCT with delayed-treatment crossover design)	−1 Risk of bias (Some concerns: small sample size, incomplete information regarding assessor blinding, and missing data for selected secondary outcomes) −2 Imprecision (n = 16; wide 95% confidence intervals; confidence interval crossed zero for the primary PUL outcome [g = +0.44, 95% CI −0.56 to +1.44] and for 3 of 5 extractable outcomes)	Very low	Significant secondary effects were observed for elbow ROM (g = +1.36, *p* = 0.018) and elbow extension strength (g = +0.94, *p* = 0.038); however, these findings derive from a single small trial and should be considered hypothesis-generating pending replication.
Engagement/motivation/fatigue perception	3 studies (k = 3): Kurt-Aydin 2024 [20] (PMOT), Baeza-Barragán 2023 [19] (EPInfant fatigue), Al-Mfarej 2023 [21] (SUES usability/relaxation)	Low (mixed study designs: 1 randomized arm-versus-arm comparison, 1 within-subject paired study, and 1 single-session feasibility study)	−1 Risk of bias (heterogeneous study designs and self-reported outcome instruments susceptible to social-desirability bias) −1 Inconsistency (different constructs assessed, including motivation, fatigue, usability, and relaxation; outcomes not directly comparable)	Very low	Direction of effect was consistently favorable across studies evaluating engagement or psychological outcomes; however, differences in outcome constructs and small sample sizes substantially limited comparability and precision. This domain demonstrated the most consistently favorable directional findings but the weakest methodological consistency.
Feasibility, safety, adherence	All 8 included studies (k = 8)	Low (predominantly observational and feasibility-oriented evidence; no studies powered to detect rare adverse events)	Risk of bias: not downgraded because the direction of effect was consistently favorable across all studies (no serious adverse events; high adherence in multi-session studies; favorable acceptance among participants, caregivers, and clinicians). Within-study limitations did not appear to materially alter the overall feasibility or safety findings. Imprecision: not downgraded for direction of effect, although small sample sizes precluded exclusion of rare adverse events (acknowledged as a limitation)	Low	This represented the highest-certainty outcome domain in the review. No serious adverse events were reported across the 8 included studies, and adherence and acceptability were consistently favorable. Nevertheless, the available sample sizes remain insufficient to exclude uncommon safety signals.
Motor learning/task-specific transfer (single-session paradigms)	4 studies (k = 4): de Freitas 2019 [13], Capelini 2017 [22], Massetti 2018 [23], Quadrado 2019 [17]	Low (single-session study designs: 3 crossover RCTs and 1 case–control study)	−1 Risk of bias (mixed-age cohorts and multiple-comparison concerns) −1 Indirectness/inconsistency (single-session task performance does not directly represent clinical motor function and may not translate to real-world performance; virtual-to-real transfer findings were inconsistent across studies, facilitated transfer in Quadrado 2019 [17] but limited transfer in Massetti 2018 [23])	Very low	Task-specific performance gains were observed; however, ecological validity remains uncertain because virtual-to-real transfer was inconsistent across studies, facilitated in Quadrado 2019 but limited in Massetti 2018 [23]. These findings should therefore be interpreted as mechanistic and hypothesis-generating rather than as evidence of clinically meaningful motor improvement.

## Data Availability

The data are available upon reasonable request from the corresponding author.

## Supplementary Materials

The following supporting information can be downloaded at: https://www.mdpi.com/article/10.3390/children13070895/s1.
