# Supplementary material for: Virtual Reality-Based Rehabilitation in Children and Adolescents with Muscular Dystrophy: A Systematic Review of Feasibility, Engagement, and Clinical Outcomes
Source: Children (Basel). 2026 Jul 3;13(7):895. doi: 10.3390/children13070895 (PMC13406484; doi:10.3390/children13070895)
Supplement: Supplementary file 1 [file children-13-00895-s001.zip › S1_Search_Strategies_revision.pdf]

Supplementary Material S1

Search Strategies

S1.1 PubMed (NCBI / NLM)

PubMed (NCBI / NLM)		
**#**	**Search expression**	**Records**
#1	"Muscular Dystrophies"[MeSH] OR "muscular dystrophy"[tiab] OR "muscular dystrophies"[tiab] OR "Duchenne"[tiab] OR DMD[tiab] OR "Becker"[tiab] OR BMD[tiab] OR "facioscapulohumeral"[tiab] OR FSHD[tiab] OR "limb-girdle"[tiab] OR LGMD[tiab] OR "Emery-Dreifuss"[tiab] OR "myotonic dystrophy"[tiab] OR "congenital muscular"[tiab]	89,997
#2	"Child"[MeSH] OR "Adolescent"[MeSH] OR "Pediatrics"[MeSH] OR child*[tiab] OR pediatric[tiab] OR paediatric[tiab] OR adolescent*[tiab] OR boy*[tiab] OR girl*[tiab] OR youth*[tiab] OR juvenile[tiab] OR teenage*[tiab] OR "school-age"[tiab]	4,880,135
#3	"Rehabilitation"[MeSH] OR "Physical Therapy Modalities"[MeSH] OR rehabilitation[tiab] OR physiotherap*[tiab] OR "physical therapy"[tiab] OR "exercise therapy"[tiab] OR "respiratory rehabilitation"[tiab] OR training[tiab] OR therap*[tiab]	3,819,999
#4	"Virtual Reality"[MeSH] OR "Video Games"[MeSH] OR "virtual reality"[tiab] OR "augmented reality"[tiab] OR "mixed reality"[tiab] OR "extended reality"[tiab] OR "VR"[tiab] OR "AR"[tiab] OR "MR"[tiab] OR exergam*[tiab] OR "serious game*"[tiab] OR "video game*"[tiab] OR Kinect[tiab] OR Wii[tiab] OR "PlayStation"[tiab] OR "head-mounted display"[tiab] OR "Oculus"[tiab] OR "Leap Motion"[tiab] OR "telerehabilitation"[tiab] OR gamif*[tiab]	56,629
**#5**	**#1 AND #2 AND #3 AND #4**	**11**

S1.2 Embase (Elsevier)

Embase (Elsevier)		
**#**	**Search expression**	**Records**
#1	'muscular dystrophy'/exp OR 'duchenne muscular dystrophy'/exp OR 'becker muscular dystrophy'/exp OR 'facioscapulohumeral dystrophy'/exp OR 'limb girdle muscular dystrophy'/exp OR 'emery dreifuss muscular dystrophy'/exp OR 'myotonic dystrophy'/exp OR 'congenital muscular dystrophy'/exp OR 'muscular dystroph*':ti,ab OR duchenne:ti,ab OR DMD:ti,ab OR becker:ti,ab OR BMD:ti,ab OR 'limb	149,131

	girdle':ti,ab OR facioscapulohumeral:ti,ab OR FSHD:ti,ab OR 'myotonic dystroph*':ti,ab	
#2	'child'/exp OR 'adolescent'/exp OR 'pediatrics'/exp OR child*:ti,ab OR pediatric:ti,ab OR paediatric:ti,ab OR adolescent*:ti,ab OR boy*:ti,ab OR girl*:ti,ab OR juvenile:ti,ab OR teenage*:ti,ab	5,705,232
#3	'rehabilitation'/exp OR 'physiotherapy'/exp OR 'kinesiotherapy'/exp OR 'exercise'/exp OR rehabilitat*:ti,ab OR physiotherap*:ti,ab OR 'physical therapy':ti,ab OR 'exercise therapy':ti,ab OR 'respiratory rehabilitation':ti,ab OR training:ti,ab	5,785,928
#4	'virtual reality'/exp OR 'augmented reality'/exp OR 'video game'/exp OR 'serious game'/exp OR 'exergaming'/exp OR 'virtual reality':ti,ab OR 'augmented reality':ti,ab OR 'mixed reality':ti,ab OR 'extended reality':ti,ab OR exergam*:ti,ab OR 'serious game*':ti,ab OR 'video game*':ti,ab OR kinect:ti,ab OR wii:ti,ab OR playstation:ti,ab OR 'head mounted display':ti,ab OR oculus:ti,ab OR 'leap motion':ti,ab OR telerehabilitat*:ti,ab	83,089
**#5**	**#1 AND #2 AND #3 AND #4**	**39**

S1.3 Cochrane Central Register of Controlled Trials (CENTRAL)

**Cochrane Central Register of Controlled Trials (CENTRAL)**		
**#**	**Search expression**	**Records**
**#1**	(MeSH descriptor: [Muscular Dystrophies] explode all trees OR ("muscular dystrophy" OR "muscular dystrophies" OR Duchenne OR DMD OR Becker OR BMD OR "facioscapulohumeral" OR FSHD OR "limb-girdle" OR LGMD OR "Emery-Dreifuss" OR "myotonic dystrophy"):ti,ab,kw) AND (MeSH descriptor: [Child] explode all trees OR MeSH descriptor: [Adolescent] explode all trees OR (child* OR pediatric OR paediatric OR adolescent* OR boy* OR girl* OR juvenile OR teenage*):ti,ab,kw) AND ("Rehabilitation" OR physiotherap* OR "physical therapy" OR "exercise therapy" OR "respiratory rehabilitation" OR training OR therap*):ti,ab,kw AND (MeSH descriptor: [Virtual Reality] explode all trees OR MeSH descriptor: [Video Games] explode all trees OR ("virtual reality" OR "augmented reality" OR "mixed reality" OR "extended reality" OR exergam* OR "serious game*" OR "video game*" OR Kinect OR Wii OR PlayStation OR Oculus OR "Leap Motion" OR telerehabilitat*):ti,ab,kw)	**17**
