# Supplementary material for: Virtual Reality-Based Rehabilitation in Children and Adolescents with Muscular Dystrophy: A Systematic Review of Feasibility, Engagement, and Clinical Outcomes
Source: Children (Basel). 2026 Jul 3;13(7):895. doi: 10.3390/children13070895 (PMC13406484; doi:10.3390/children13070895)
Supplement: Supplementary file 1 [file children-13-00895-s001.zip › S2_PRISMA_2020_Checklist_revision.pdf]

Supplementary Material S2

PRISMA 2020 Checklist

#	Item	Checklist item	Location in manuscript / Supplementary
**Title**			
1	**Title**	Identify the report as a systematic review.	Title page: "Virtual Reality-Based Rehabilitation in Pediatric Muscular Dystrophy: A Systematic Review and Structured Narrative Synthesis"
**Abstract**			
2	**Abstract**	See PRISMA 2020 for Abstracts checklist.	Abstract structured into Background, Objectives, Methods, Results, Conclusions; PRISMA 2020 compliance noted.
**Introduction**			
3	**Rationale**	Describe the rationale for the review in the context of existing knowledge.	Section 1.1–1.4 (Disease background → conventional rehab limitations → VR rationale → evidence gap)
4	**Objectives**	Provide an explicit statement of the objective(s) or question(s) the review addresses.	Section 1.5 (four explicit objectives: identify/characterize, feasibility/safety, engagement/motor outcomes, propose stage-based framework)
**Methods**			
5	**Eligibility criteria**	Specify the inclusion and exclusion criteria for the review and how studies were grouped for the syntheses.	Section 2.2 (PICOS-T: Population, Intervention, Comparator, Outcomes, Study design + Time/language). Studies grouped by disease stage in synthesis (Section 2.6).
6	**Information sources**	Specify all databases, registers, websites, organisations, reference lists and other sources searched or consulted.	Section 2.3 (PubMed, Embase, Cochrane CENTRAL; date searched 2026-04-21). Detailed sources in Supplementary Material 1.
7	**Search strategy**	Present the full search strategies for all databases, registers and websites, including any filters and limits used.	Section 2.3 (concept structure summarized). Full search strings in Supplementary Material 1 (S1.1–S1.3).
8	**Selection process**	Specify the methods used to decide whether a study met the inclusion criteria.	Section 2.4 (dual-reviewer T/A screening; full-text dual review; Cohen's κ = 0.897; consensus resolution; Q1–Q4 amendments).
9	**Data collection process**	Specify the methods used to collect data from reports.	Section 2.5 (standardized 14-sheet extraction

#	Item	Checklist item	Location in manuscript / Supplementary
			template; single-reviewer extraction with full re-verification; QA Corrections Log).
**10a**	**Data items (outcomes)**	List and define all outcomes for which data were sought.	Section 2.2 (motor function, upper-limb function, ADL, QoL, adherence, motivation, safety) and Section 2.6 (five outcome domains for GRADE).
**10b**	**Data items (other)**	List and define all other variables for which data were sought.	Section 2.5 (study characteristics, population, intervention, comparator, outcome instruments, AEs, adherence, RoB).
**11**	**Study risk of bias assessment**	Specify the methods used to assess risk of bias in the included studies.	Section 2.5 (RoB 2 and ROBINS-I design-appropriate tools). Dual-reviewer assessment with 100% concordance (Cohen's $\kappa = 1.0$). Table 2.
**12**	**Effect measures**	Specify for each outcome the effect measure(s) used in the synthesis or presentation of results.	Section 2.6 (direction of effect; descriptive single-study Hedges' g for Heutinck 2018 reported descriptively in Results, not pooled).
**13a**	**Synthesis methods (eligibility)**	Describe the processes used to decide which studies were eligible for each synthesis.	Section 2.6 (stage-based grouping grounded in the Vignos lower-extremity functional scale and supporting clinical characteristics; outcome-domain grouping for GRADE).
**13b**	**Synthesis methods (preparation)**	Describe any methods required to prepare the data for presentation or synthesis.	Section 2.5 (data extraction template; numerical re-verification; READY/CAUTION/HOLD/DO NOT POOL labels).
**13c**	**Synthesis methods (visual)**	Describe any methods used to tabulate or visually display results.	Section 2.6 (structured narrative synthesis); Table 3 (GRADE); Figure 2 (stage-based synthesis).
**13d**	**Synthesis methods (statistical)**	Describe any methods used to synthesize results and provide a rationale for any decisions used.	Section 2.6 (no meta-analysis; rationale: clinical, methodological, and outcome heterogeneity); Discussion 4.2 (Feasibility and safety as the strongest evidence).
**13e**	**Synthesis methods (heterogeneity)**	Describe any methods used to explore possible causes of heterogeneity.	Section 2.6 (heterogeneity itself was the organizing principle — disease stage as primary axis); Section 3.3 (Stage-Based Synthesis).
**13f**	**Synthesis methods (sensitivity)**	Describe any sensitivity analyses conducted.	Not applicable: no quantitative pooling was performed. Stage-based synthesis serves as the structured exploration of methodological heterogeneity.
**14**	**Reporting bias assessment**	Describe any methods used to assess risk of bias due to missing results in a synthesis.	Section 2.5 (D5 selection of reported result, in RoB 2 and ROBINS-I domains); Section 3.4 (publication bias not formally assessed given k=7); Section 4.6 (Strengths and Limitations: small sample limit precludes funnel-plot analysis).

#	Item	Checklist item	Location in manuscript / Supplementary
15	**Certainty assessment**	Describe any methods used to assess certainty (or confidence) in the body of evidence for an outcome.	Section 2.6 (GRADE approach at outcome-domain level); Table 3 (GRADE evidence profile).
**Results**			
16a	**Study selection (numbers)**	Describe the results of the search and selection process from the number of records identified to the number of studies included in the review.	Section 3.1 (search, screening, and inclusion summary: 67 records → 8 included). Figure 1 (PRISMA 2020 flow diagram with all stage counts).
16b	**Study selection (excluded)**	Cite studies that might appear to meet the inclusion criteria but were excluded, with reasons.	Section 3.1 (4 full-text exclusions). Reasons summarized in Figure 1 (PRISMA 2020 flow diagram).
17	**Study characteristics**	Cite each included study and present its characteristics.	Section 3.1; Table 1 (Study characteristics — design, country, n, VR platform, intervention, comparator, primary outcome).
18	**Risk of bias in studies**	Present assessments of risk of bias for each included study.	Section 3.2; Table 2 (8 evidence rows for 8 studies — Kurt-Aydin split into VR-vs-Biofeedback and VR-vs-Control contrasts).
19	**Results of individual studies**	For all outcomes, present, for each study: summary statistics for each group and an effect estimate and its precision.	Section 3.3; descriptive single-study Hedges' g with 95% CI for Heutinck 2018 reported in Section 3.3.2 prose.
20a	**Results of syntheses (a)**	For each synthesis, briefly summarise the characteristics and risk of bias among contributing studies.	Section 3.3 (stage-based synthesis); Table 1 (study characteristics) and Table 2 (RoB) reported per study.
20b	**Results of syntheses (b)**	Present results of all statistical syntheses conducted.	Not applicable (no statistical pooling). Section 3.3 (structured narrative synthesis by stage).
20c	**Results of syntheses (c)**	Present results of all investigations of possible causes of heterogeneity.	Section 3.3 (stage-based organization is itself the heterogeneity exploration).
20d	**Results of syntheses (d)**	Present results of all sensitivity analyses conducted.	Not applicable; no pooled estimates to test for sensitivity.
21	**Reporting biases**	Present assessments of risk of bias due to missing results for each synthesis assessed.	Section 3.2 (D5 of RoB tools); Section 3.4 (publication bias not formally assessed); Section 4.6 (Strengths and Limitations).
22	**Certainty of evidence**	Present assessments of certainty (or confidence) in the body of evidence for each outcome assessed.	Section 3.4 (Certainty of Evidence — GRADE); Table 3 (Very low for 4 of 5 domains; Low for feasibility/safety/adherence).
**Discussion**			
23a	**Discussion (general)**	Provide a general interpretation of the results in the context of other evidence.	Section 4.1 (Principal Findings); Section 4.5 (Stage-Based Framework as conceptual contribution);

#	Item	Checklist item	Location in manuscript / Supplementary
			comparison with Baeza-Barragán 2020, Kiper 2024.
**23b**	**Discussion (limitations of evidence)**	Discuss any limitations of the evidence included in the review.	Section 4.2 (Feasibility and safety as the strongest evidence); Section 4.4 (motor outcome heterogeneity); Section 4.6 (Limitations: small k, indirectness, English-only, IEEE Xplore).
**23c**	**Discussion (limitations of process)**	Discuss any limitations of the review processes used.	Section 4.6 (mixed-age cohorts; biomedical-only databases; protocol amendments documented; RoB dual-reviewer).
**23d**	**Discussion (implications)**	Discuss implications of the results for practice, policy, and future research.	Section 4.5 (stage-based clinical implications); Section 4.7 (Implications for Future Research and Clinical Practice).
**Other information**			
**24a**	**Registration and protocol (a)**	Provide registration information for the review, including register name and registration number.	Section 2.1 (PROSPERO ID CRD420261380539).
**24b**	**Registration and protocol (b)**	Indicate where the review protocol can be accessed, or state that a protocol was not prepared.	Section 2.1 (a priori protocol; PROSPERO public record). Methods 2.4 (Q1–Q4 amendments described).
**24c**	**Registration and protocol (c)**	Describe and explain any amendments to information provided at registration or in the protocol.	Section 2.4 (Q1–Q4 amendments summarised in the manuscript).
**25**	**Support**	Describe sources of financial or non-financial support, and the role of the funders or sponsors.	This study was supported by the National Research Foundation of Korea (NRF-RS-2025-2468362440982119420001) and a grant (BCRI-123021) from Chonnam National University Hospital Biomedical Research Institute. The funders had no role in study design; collection, analysis, or interpretation of data; writing of the report; or the decision to submit for publication.
**26**	**Competing interests**	Declare any competing interests of review authors.	All authors declare that they have no conflicts of interest relevant to this work.
**27**	**Availability of data, code and other materials**	Report which of the following are publicly available and where: template data collection forms; data extracted from included studies; data used for all analyses; analytic code; any other materials used in the review.	Data extraction master file (Data_Extraction_AllStudies_v2.xlsx, 15 sheets) available on request from the corresponding author. Search strategies (S1) and PRISMA 2020 checklist (S2) are provided as Supplementary Materials.
